# Sajous's Annual and Analytical Cyclopædia of Practical Medicine

**Published:** 1900-03

**Authors:** 


**Sajous's Annual and Analytical Cyclopædia of Practical Medicine.** Vol. IV. *Infants, Diarrhœal Diseases of*, to *Mercury*. Pp. vii., 622. Philadelphia: The F. A. Davis Company. 1899.

The Editor in his preface directs especial attention to the first article by Professor Blackader, of Montreal, and to an elaborate paper on Malarial Fevers, by Professor James C. Wilson and Dr. Thomas G. Ashton. The article on Locomotor Ataxia, by Dr. W. B. Pritchard, of New York; that on Intubation, by Professor F. E. Waxham, of Chicago; and that on Diseases of the Liver, by Professor Alexander McPhedran, of Toronto; that on Meningitis, by Dr. Charles M. Hay, of Philadelphia, are all entitled to special notice as models of their kind. The article on Insanity was one of the last works of Professor George H. Rohé, of Baltimore, who had been connected with the *Annual* since 1891. This article "is in keeping with the sincerity of purpose that characterized him."

